# Bioassay tests reveal for the first time pyrethroid resistance in *Aedes* mosquitoes from Franceville, southeast Gabon, Central Africa

**DOI:** 10.1051/parasite/2025036

**Published:** 2025-07-01

**Authors:** Judicaël Obame-Nkoghe, Faël Moudoumi Kondji, El Hadji Diouf, Omar Thiaw, Brad Ghaven Niangui, Arnauld Ondo-Oyono, Yasmine Okomo-Nguema, Neil Michel Longo-Pendy, Franck Mounioko, Boris Makanga, Basile Kamgang, Christophe Paupy, Pierre Kengne, Patricks Voua Otomo, El Hadji Amadou Niang

**Affiliations:** 1 Département de Biologie de l’Université des Sciences et Techniques de Masuku (USTM) BP 901 Franceville Gabon; 2 Unité de Recherche en Ecologie de la Santé du Centre Interdisciplinaire de Recherches Médicales de Franceville (CIRMF) BP 769 Franceville Gabon; 3 Department of Zoology and Entomology, Faculty of Natural and Agricultural Sciences, University of the Free State Phuthaditjhaba 9866 Republic of South Africa; 4 Laboratoire d’Écologie Vectorielle et Parasitaire, Université Cheikh Anta Diop de Dakar BP 5005 Dakar Sénégal; 5 Research Institute in Tropical Ecology (IRET) BP 13354 Libreville Gabon; 6 Centre for Research in Infectious Diseases (CRID) PO Box 13591 Yaoundé Cameroun; 7 MIVEGEC, Univ. Montpellier, CNRS, IRD 34394 Montpellier France

**Keywords:** *Aedes*, Insecticide resistance, Pyrethroids, Organophosphates, Carbamates, PBO

## Abstract

The spread of resistance to insecticides, such as pyrethroids, in *Aedes* vectors increases the risk of spread of arboviral diseases. In Gabon, the insecticide resistance profiles of *Ae. aegypti* and *Ae. albopictus* species remain poorly known. During a study to monitor the dynamics of *Aedes* populations in Franceville, in south-east Gabon, the resistance profiles of these two species to pyrethroids, organophosphates and carbamates were assessed. Susceptibility tests on adults and synergist tests with piperonyl butoxide (PBO) were carried out as per the World Health Organization protocol. The results showed that *Ae. aegypti* and *Ae. albopictus* were susceptible to permethrin, pirimiphos-methyl and bendiocarb. However, both species were resistant to deltamethrin (mortality: 67% for *Ae. aegypti*; 33% for *Ae. albopictus*). Exposure to a 5-fold dose of deltamethrin increased mortality to 100% and 91% for *Ae. aegypti* and *Ae. albopictus*, respectively. Resistance to alpha-cypermethrin was also recorded (mortality: 82% for *Ae. aegypti*; 64.6% for *Ae. albopictus*). Pre-exposure to PBO resulted in the restoration of susceptibility to deltamethrin and alpha-cypermethrin for *Ae. aegypti*, and a significant increase in mortality for *Ae. albopictus*. These data provide the first evidence of pyrethroid resistance in *Aedes* in Gabon and could help to establish more effective control measures against arbovirus vectors.

## Abstract (fr)

La propagation de la résistance aux insecticides, tels que les pyréthrinoïdes, chez les vecteurs *Aedes* augmente le risque de propagation des arboviroses. Au Gabon, les profils de résistance aux insecticides des espèces *Ae. aegypti* et *Ae. albopictus* restent mal connus. Lors d’une étude de suivi de la dynamique des populations d’*Aedes* à Franceville, dans le sud-est du Gabon, les profils de résistance de ces deux espèces aux pyréthrinoïdes, aux organophosphorés et aux carbamates ont été évalués. Des tests de sensibilité sur adultes et des tests synergétiques au butoxyde de pipéronyle (PBO) ont été réalisés conformément au protocole de l’Organisation Mondiale de la Santé. Les résultats ont montré qu’*Ae. aegypti* et *Ae. albopictus* étaient sensibles à la perméthrine, au pirimiphos-méthyl et au bendiocarbe. Cependant, les deux espèces étaient résistantes à la deltaméthrine (mortalité : 67 % pour *Ae. aegypti*, 33 % pour *Ae. albopictus*). L’exposition à une dose de deltaméthrine multipliée par 5 a augmenté la mortalité, respectivement à 100 % pour *Ae. aegypti* et 91 % et pour *Ae. albopictus*. Une résistance à l’alpha-cyperméthrine a également été observée (mortalité : 82 % pour *Ae. aegypti*, 64,6 % pour *Ae. albopictus*). Une préexposition au PBO a entraîné la restauration de la sensibilité à la deltaméthrine et à l’alpha-cyperméthrine pour *Ae. aegypti*, et une augmentation significative de la mortalité pour *Ae. albopictus*. Ces données fournissent la première preuve de résistance aux pyréthroïdes chez les *Aedes* au Gabon et pourraient aider à établir des mesures de contrôle plus efficaces contre les vecteurs d’arbovirus.

## Introduction

Arboviruses (arthropod-borne viruses) are vector-borne pathogens transmitted by blood-sucking arthropods such as mosquitoes, ticks, and sandflies [39]. The diseases they can cause include dengue fever, chikungunya, Zika, and yellow fever, which pose a global health threat. These diseases, confined to tropical and sub-tropical regions for a long time, are now affecting temperate areas [8, 11, 34]. The spread of arboviruses is closely linked to the geographical expansion of their vectors, particularly *Aedes* mosquitoes, among which *Aedes aegypti* (Linnaeus, 1762) and *Aedes albopictus* (Skuse, 1894) are the main vector species*.*

*Aedes aegypti* is a species native to Africa, and known to be the main vector of dengue fever throughout the world [16]. *Aedes albopictus*, originating from Asia, has succeeded in colonizing other continents during the past four decades [30] through the development of human activities such as the international trade of used tires [30, 40]. In Central Africa, the invasion by *Ae. albopictus* has been found to be associated with numerous outbreaks or silent epidemic waves of chikungunya, dengue, and Zika [1, 31, 33].

Given the absence of any specific curative treatment or effective vaccine that can be used in the general population (except for the yellow fever virus), vector control is the key strategy for reducing the risks posed by the vector-borne transmission of these arboviruses. The World Health Organization (WHO) recommends the use of insecticides during outbreaks [44]. However, the emergence of resistance to the most commonly used insecticides is a real obstacle to the progress made to date [25], and on a global scale, mosquito resistance to insecticides represents a major challenge for vector control programmes. Various studies across Africa [7], Asia [41, 47], and Latin America [38] have shown worrying trends in insecticide resistance, requiring global as well as context-adapted approaches to develop sustainable management strategies.

In Gabon, due to the heavy burden of endemic malaria in the region, the use of insecticides mainly targets *Anopheles* mosquitoes to reduce the transmission of this disease. Several studies have been carried out across the country to characterize the resistance profile of *Anopheles* mosquitoes to the main families of insecticides (pyrethroids, carbamates, and organophosphates) [4, 5, 27, 32]. Conversely, among *Aedes*, despite the endemic circulation of dengue fever and chikungunya epidemics recorded in the country in 2007 and 2010 [15, 29, 31], very little insecticide resistance monitoring and control data on *Ae. albopictus* and *Ae. aegypti* populations are currently available [19].

To improve our knowledge of insecticide resistance in *Ae. albopictus* and *Ae. aegypti* in Gabon, we conducted an exploratory study alongside a broader survey on mosquito population dynamics in southeastern Gabon. This study aimed to address the existing gap by assessing the resistance profiles of these two vector species to pyrethroids and other insecticide classes, including organophosphates and carbamates in Franceville, southeastern Gabon.

## Materials and methods

### Ethics

All mosquito collections were conducted in accordance with national regulations and with permission from local authorities under the number N°AR33/23/MESRSIT/CENAREST/CG/CST/CSAR.

### Study area

**Figure 1:**
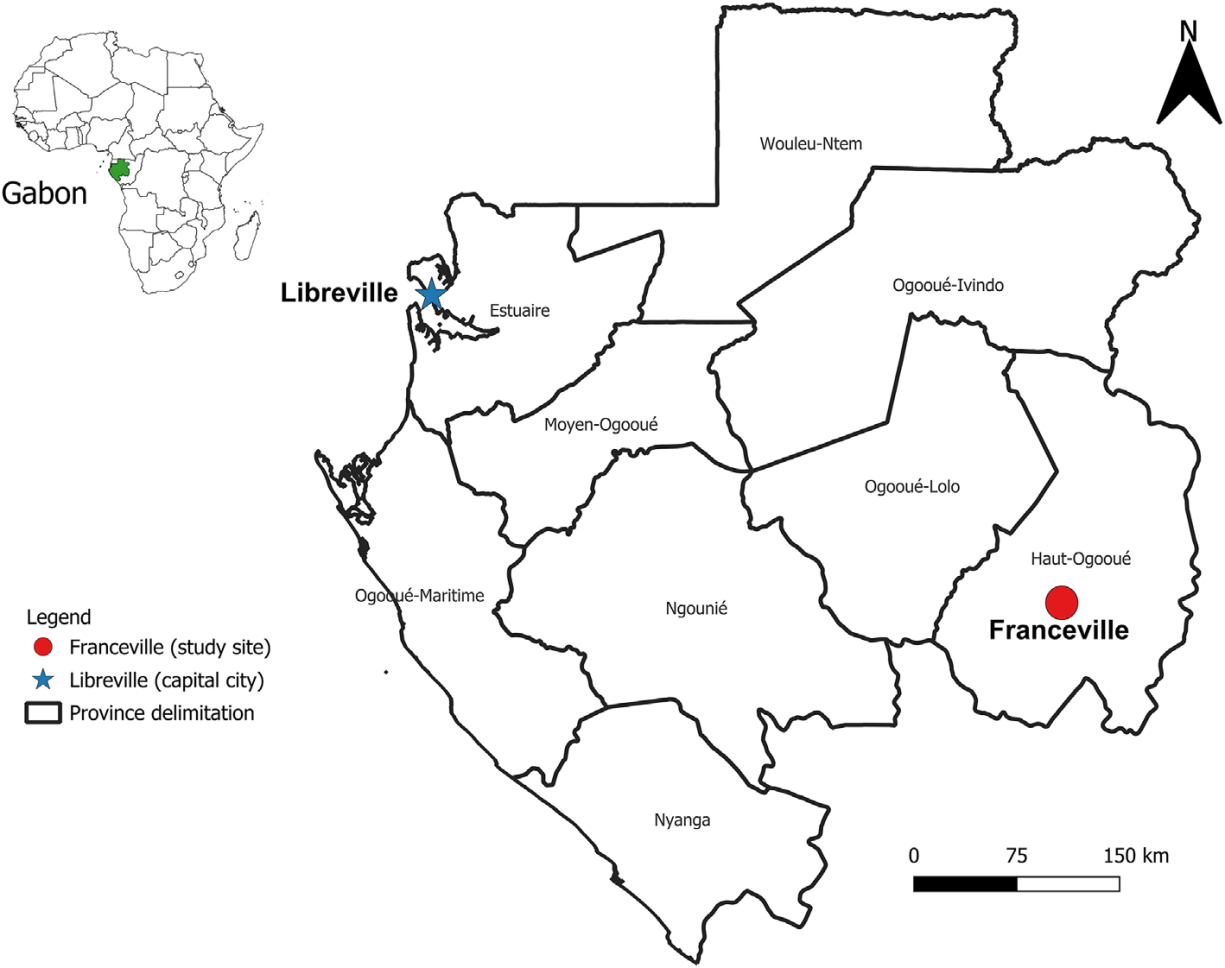
Localisation of Franceville.

This study was conducted in Franceville (S01°37′15″, E13°34′58″), located in the Haut-Ogooué province in southeastern Gabon (Fig. 1). Situated over 600 km from Libreville, the capital city, Franceville is the third most populous city in Gabon, following Libreville and Port-Gentil. Franceville is characterized by an equatorial climate featuring two rainy seasons (a longer one from March to June and a shorter one from October to November) and two intervening dry seasons. Franceville is divided into four districts, each marked by a semi-urban environment. Over the past two decades, the city has experienced outbreaks of dengue and chikungunya, ranking it among the most affected areas in the country, and hosts a high density of *Aedes* mosquitoes. However, until today, no vector control programmes have been considered, especially given the need to document the status of resistance to insecticides to inform potential future control measures.

### Mosquito sampling

The sampling occurred between March and June 2023, during the rainy season. We used G0 adult stages that emerged from field-collected eggs or larvae to perform the bioassays. Collections of eggs and larvae were conducted using randomly placed ovitraps in domestic and peri-domestic sites across the four districts of Franceville (2–3 per site). Opportunistic inspections of domestic or discarded water containers were conducted in the same zones to gather *Aedes* immature stages (larvae and pupae). To obtain a general overview of resistance patterns in Franceville, we used composite populations from the four districts for each species. In each district, we deployed 10–15 plastic ovitraps [spaced 20 m apart to reduce potential competition effects] to collect eggs, while larvae and pupae were sampled from various water containers [30–35 per district], including flower pots, cinder blocks, and discarded tires. Each ovitrap consisted of a black plastic cup filled with up to 300 mL of tap water and a 15 cm × 5 cm wooden strip, which served as an oviposition substrate. The wooden strips were removed every 5 to 7 days and taken to the insectary for drying and subsequent egg hatching. The dried eggs (representing the G1 generation) were then placed in plastic trays containing 1 L of distilled water, where they were hatched and reared to the adult stage. Larvae collected from each site (also representing the G0 generation) were preserved in 500 mL plastic containers and transported to the insectary. There, they were transferred into 30 cm × 15 cm plastic rearing trays until the adult stage. In cases where field-collected water volume was insufficient, we supplemented with up to 1 L of distilled water. Larvae were fed daily with 1–2 g of Tetramin baby^®^ fish food. All trays were maintained under room conditions: temperature 27 °C ± 2 °C, relative humidity 80% ± 10%, and a 12:12 h (light:dark) photoperiod. Upon emergence, adults were individually introduced into a dry hemolysis tube plugged with a cotton ball, and morphologically identified using a binocular microscope and “customized” taxonomic keys based on the updates of Edwards’ identification keys for Ethiopian mosquitoes [13] and Huang’s key for the subgenus *Stegomyia* of *Aedes* mosquitoes from the Afrotropical region [17]. After identification, adults *Ae. albopictus* and *Ae. aegypti* coming from each district were pooled by species in cages and fed exclusively on 10% sucrose. In order to ensure synchronised age of mosquitoes, new cages were used to pool emerging adults every 5 days. They were maintained at insectary conditions, as indicated earlier, until tests.

### Susceptibility and synergist tests

Bioassays were performed on adult female mosquitoes obtained from eggs and larvae to assess the phenotypic resistance of *Ae. albopictus* and *Ae. aegypti* to insecticide molecules according to the World Health Organization (WHO) tube test protocol [44]. A strain of *Ae. albopictus* from the remote forested village of Kessipougou (S00°54′27.7″, E012°47′36.5″), presumed pesticide-naïve, was used as the susceptible reference strain in these tests. In Kessipougou, most residents report not using insecticides, and eggs were collected along forest interfaces at about 30 m of the nearest house using 10 ovitraps (5 at each side of the village) activated simultaneously for 5 days and positioned at least 30 m from each other. Before testing Franceville mosquitoes, we conducted preliminary assays to validate insecticide efficacy. No prior published resistance data were available for the Kessipougou strain, but our results confirmed the assumption of the strain’s full susceptibility, establishing its suitability as a control reference. The impregnated papers used in the bioassays were sourced from the Vector Control Research Unit (VCRU) at the University of Sains, Malaysia. The VCRU is the WHO collaborating center for the manufacturing of insecticide-impregnated papers. Five insecticide compounds from three major classes (pyrethroids, organophosphates, and carbamates) were used in the tests. The discriminating concentrations (DCs) used in this study for testing *Aedes* mosquitoes were those available from the VCRU and recommended by the WHO before the latest adopted DCs. Specifically, the pyrethroids included 0.75% permethrin (later revised to 0.4%), 0.05% deltamethrin (later revised to 0.03%), and 0.05% alpha-cypermethrin (later revised to 0.08% for *Ae. albopictus*, but maintained at 0.05% for *Ae. aegypti*). The organophosphates comprised 0.25% pirimiphos-methyl (later revised to 60 mg/m^2^, equivalent to 0.15%), while the carbamates included 0.1% bendiocarb (later revised to 0.2%). These DCs were those available at the time of the study. Insecticide-free papers were used for control tests with silicone oil as solvent for the tests with pyrethroids, or olive oil for the tests with organophosphates and carbamates. For each insecticide DC, four test replicates (insecticide-impregnated papers) and two controls (insecticide-free papers) were used. For each replicate, 25 G1 non-blood-fed females aged between 2 and 5 days were introduced into exposure tubes containing insecticide-impregnated filter papers for 1 h and knockdown (Kd) was recorded for each pyrethroid. Post-exposed mosquitoes were transferred into holding tubes and kept at the insectary conditions with free access to 10% sucrose, and mortality was recorded 24 h post-exposure. Only when resistance to a given insecticide was confirmed four replicates at 5-fold of the initial dose were performed to assess the intensity of the observed resistance. However, this was not possible for alpha-cypermethrin given that 5-fold doses (0.25%) were not available for testing.

To assess the potential role of metabolic resistance mechanisms, synergistic tests with 4% piperonyl butoxide (PBO) were performed on G1 adult females aged between 2 and 5 days. PBO works by inhibiting certain metabolic detoxifying enzymes such as cytochrome P450 monooxygenases (P450s), which are involved in the metabolic neutralization of insecticide compounds, especially pyrethroids [3]. To process the tests, females were pre-exposed for 1 hour to papers impregnated with PBO, then immediately exposed to the selected insecticides (or insecticide-free papers for controls), following the procedure described above for standard tests.

### Data analysis

Interpretation of mortality rates was based on the WHO criteria. Mosquitoes were considered resistant when mortality was less than 90%, and susceptible when mortality was greater than or equal to 98%. However, when mortality was greater than or equal to 90% and less than 98%, resistance to the insecticide tested was suspected. Corrections were applied when necessary, taking into account the mortality in controls using Abbott’s formula [44]. R software, version 4.3.3, was used to produce the graphical representations and to perform appropriate statistical tests. The chi-squared test was used to compare the mortality percentage between *Ae. albopictus* and *Ae. aegypti* for each insecticide. When chi-squared test conditions were not met, Fisher’s exact test was used as a non-parametric alternative. All differences were considered statistically significant when the *p*-value was below 0.05.

## Results

### Phenotypic insecticide resistance profile

**Figure 2:**
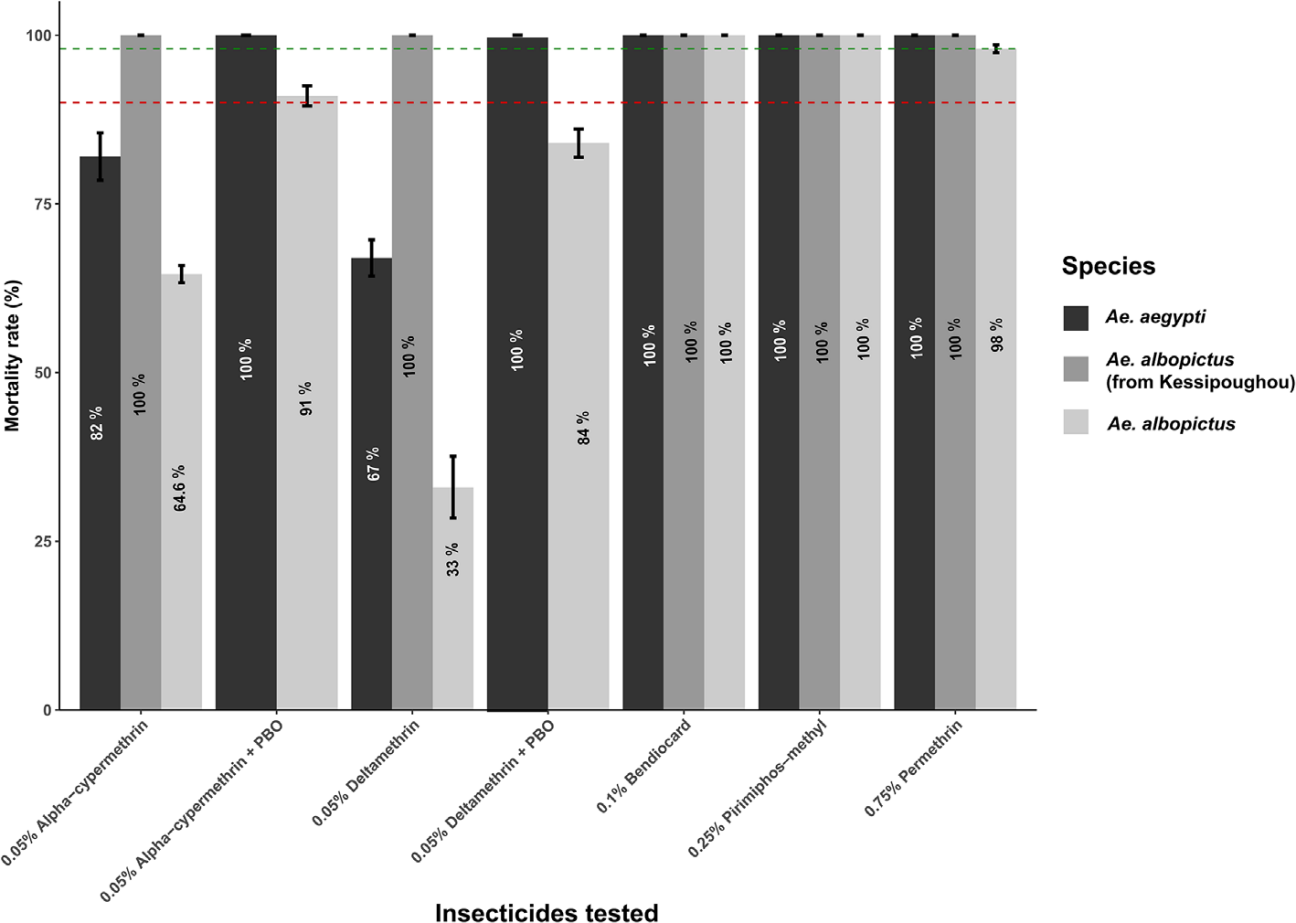
Mortality of adult females 24 h after 1-hour exposure to insecticides and pre-exposure to PBO. The red dotted line indicates 90% mortality, and the green one 98% mortality*.*

A total of 1,600 female mosquitoes (800 *Ae. albopictus* and 800 *Ae. aegypti*) were exposed to insecticides. Results showed that all *Ae. albopictus* and *Ae. aegypti* females were knocked down after 60 min of exposure to all pyrethroid insecticides. *Aedes albopictus* from the remote forested village of Kessipougou had 100% mortality for all insecticides tested. Exposure of mosquitoes to bendiocarb (carbamate) and pirimiphos-methyl (organophosphate) resulted in 100% mortality rates for both *Ae. albopictus* and *Ae. aegypti*. In contrast, exposure to pyrethroids resulted in variable mortality rates. *Aedes albopictus* was susceptible to permethrin (98% mortality), but resistant to deltamethrin (33%) and alpha-cypermethrin (64.6%). *Aedes aegypti* was susceptible to permethrin, but resistant to deltamethrin (71%) and alpha-cypermethrin (82%) (Fig. 2). Results on intensity testing demonstrated that deltamethrin resistance was low in *Ae. aegypti*, but moderate in *Ae. albopictus* populations, with mortality rates of 100% and 91% at 5X standard dose, respectively.

Statistical analysis showed that the mortality rates recorded after exposure to alpha-cypermethrin 0.05%, deltamethrin 0.05%, and deltamethrin 0.25% were significantly higher in *Ae. aegypti* compared to *Ae. albopictus* (Table 1).

**Table 1:** Comparison of mortality rates in *Ae. aegypti* vs. *Ae. albopictus.*

Insecticides	Mortality %		*χ* ^2^	df	*p*-value
	*Ae. aegypti*	*Ae. albopictus*			
0.05% alphacypermethrin	82	64.6	6.6	1	0.01
0.05% deltamethrin	67	33	43.6	1	<0.001
0.25% deltamethrin	100	90	8.5	1	0.003
0.75% permethrin	100	98	–	–	0.5*
0.1% bendiocarb	100	100	–	–	1*
0.25% pirimiphos-methyl	100	100	–	–	1*

* Fisher test.

### Assessment of potential resistance mechanisms

The results of bioassays with the synergist PBO in *Ae. albopictus* showed a partial recovery of susceptibility to 0.05% deltamethrin (from 33% mortality without pre-exposure to PBO to 84% mortality after 60 min pre-exposure to PBO, *χ*^2^ = 50.6, df = 1, *p* < 0.001), and to 0.05% alpha-cypermethrin (from 64.6% mortality without pre-exposure to PBO to 91% mortality after 60 min pre-exposure to PBO, *χ*^2^ = 18.2, df = 1, *p* < 0.001). In *Ae. aegypti*, pre-exposure to PBO resulted in a complete recovery of susceptibility to 0.05% deltamethrin (from 67% mortality without pre-exposure to PBO to 100% mortality after pre-exposure to PBO, Fisher *p* < 0.001), and to 0.05% alpha-cypermethrin (from 82% mortality without pre-exposure to PBO to 100% mortality after pre-exposure to PBO, Fisher *p* < 0.001) (Fig. 2). This partial or complete recovery of susceptibility suggests the probable involvement of active metabolic detoxification enzymes, particularly cytochrome P450 monooxygenases, in pyrethroid resistance in both species.

## Discussion

To date, limited research has been conducted in monitoring insecticide resistance in *Aedes* mosquitoes, particularly *Ae. albopictus* and *Ae. aegypti*, across Africa. According to recent studies spanning the past two decades and encompassing 18 African countries [14, 18, 24, 26, 28, 36, 43, 46], widespread confirmed or suspected resistance to DDT (dichlorodiphenyltrichloroethane) has been documented in both species. In contrast, resistance to pyrethroids remains less evident and appears to be an emerging concern, with a higher prevalence reported in *Ae. aegypti* (recorded in 16 out of the 18 countries) compared to *Ae. albopictus* (detected in only 3 countries). A similar pattern was observed for carbamate or organophosphate insecticides (9/18 for *Ae. aegypti* versus 2/18 for *Ae. albopictus*) [14, 43]. In Gabon specifically, resistance assessments in *Aedes* mosquitoes have been restricted to temephos for larval stages, and to DDT, deltamethrin, propoxur, and fenitrothion for adults, with confirmed resistance in adult *Ae. aegypti* to DDT [19].

The present study aimed to characterise the phenotypic profile of insecticide resistance of the species *Ae. albopictus* and *Ae. aegypti* in southeastern Gabon. The observed mortality rates from exposure tests with pirimiphos-methyl (organophosphate) and bendiocarb (carbamate) indicate that both species are susceptible to these insecticides. These findings are consistent with those reported in southern Benin (West Africa), where populations of *Ae. aegypti* were shown to be susceptible to both insecticides [21]. Similar susceptibility to bendiocarb has also been documented in Cameroon (Central Africa) for populations of both *Ae. albopictus* and *Ae. aegypti* [12, 20, 26]*.* In both studies (Benin and Cameroon), pirimiphos-methyl and bendiocarb were used at concentrations similar to those used in our investigation in Gabon. There could be similar operational strategies in these African countries, where the use of carbamates and organophosphates is more controlled and maintains effectiveness. However, these results contrast with findings from Asia, particularly in Singapore, where populations of *Ae. albopictus* have been reported as resistant to pirimiphos-methyl, with mortality rates ranging from 45% to 75% [23]. Indeed, Lee *et al.* suggested the long-term implementation of dengue control using pirimiphos-methyl for vector control in Singapore as a promoting factor of the resistance of *Ae. albopictus* populations. Therefore, operational differences in both African and Asian contexts may explain the contrasting results. In the context of vector control strategies, maintaining low or reduced selection pressure on insecticide-resistant mosquito populations through controlled, reduced, or minimal use of a specific insecticide may help preserve or restore their susceptibility [6]. Therefore, the results that we observed could be attributed, in part, to the fact that pirimiphos-methyl and bendiocarb are not employed in public health initiatives in Gabon. Additionally, their limited use in agriculture, as suggested by a similar study on *Anopheles* mosquitoes in Mouila, southern Gabon [22], may also explain these findings.

Exposure to pyrethroids showed that both *Ae. albopictus* and *Ae. aegypti* were susceptible to 0.75% permethrin, but resistant to 0.05% deltamethrin and 0.05% alpha-cypermethrin. These findings represent the first documented evidence of resistance to alpha-cypermethrin and susceptibility to permethrin in these species. However, the results for deltamethrin contrast with earlier data from Libreville, north-western Gabon, where *Ae. aegypti* was reported as susceptible to a slightly higher concentration (0.06%) nearly 15 years ago [19]. Collectively, these results provide the first confirmed evidence of pyrethroid resistance in *Aedes* mosquitoes in Gabon. The earlier susceptibility could have resulted from a lower selection pressure than the present study period, such as the less vulgarised use of pesticides in urban market gardening, known as a key driver in resistance [2]. Pyrethroid resistance in *Aedes* mosquitoes has been documented across multiple African regions [14, 43]. For example, *Ae. albopictus* and *Ae. aegypti* in Cameroon were found to be resistant to permethrin [46], as well as to deltamethrin and alpha-cypermethrin [26, 45]. While the prevalence of pyrethroid resistance is increasing, the underlying mechanisms remain incompletely understood. One potential driver is the extensive and often unregulated application of pyrethroids for malaria vector control, which exerts substantial selection pressure on mosquito populations [2], including non-target species. Additionally, the widespread use of pyrethroid-based pesticides in agricultural practices may further accelerate the development of resistance in *Aedes* populations [45]. In Franceville, malaria vector control is poorly implemented. However, urban vegetable farming is common, and the use of pesticides by farmers is poorly regulated, making it a potential indirect source of resistance selection pressure.

We showed that mortality rates for deltamethrin and alpha-cypermethrin were significantly higher in the native species *Ae. aegypti* compared to the invasive *Ae. albopictus*. One possible explanation may be that *Ae. albopictus* possesses greater innate resistance potential (genetic traits) or adaptive capacity (genetic and/or environmental factors) to these insecticides compared to *Ae. aegypti*. This further highlights the need to monitor in particular the spatiotemporal dynamics of insecticide resistance for this newly introduced species in the country, where it is an active vector of arboviruses [31], to better anticipate appropriate control strategies.

Pre-exposure to PBO fully restored susceptibility to deltamethrin and alpha-cypermethrin in *Ae. aegypti*, and significantly increased mortality in *Ae. albopictus*. This could suggest that the cytochrome P450 monooxygenase enzymes play the main role in the observed phenotypic resistance [10, 26].

We observed that all the mosquitoes tested were susceptible to the knockdown (Kd) effect. It is known that mutations such as F1534C, V1016G, V1016I, S989P, or L1014F in the voltage-gated sodium channel (vgsc) gene are associated with Kd resistance in *Aedes* mosquitoes [9, 38]. Thus, in the absence of molecular investigations, our phenotypic results may suggest the absence of these target-site resistance mutations in *Ae. aegypti* and *Ae. albopictus*, although this cannot be confirmed without genotypic analysis. Our observations are nonetheless broadly consistent with findings observed in *Ae. aegypti* populations from Senegal [37]. Therefore, from a public health strategy perspective, one of the solutions likely to restore insecticide susceptibility in these vectors in case they are resistant could be a more strategic and appropriate regulation of insecticide/pesticide use, integrating synergists in formulations to bypass the metabolic mechanisms that might be involved in resistance. Moreover, the efficacy of bendiocarb and pirimiphos-methyl observed in *Ae. albopictus* and *Ae. aegypti* makes these insecticides reliable alternatives to pyrethroids in Gabon. However, given their associated toxic effects on non-target organisms [42], they will require controlled use in case of emergencies.

## Conclusion

This exploratory study provides the first evidence of pyrethroid resistance in *Ae. albopictus* and *Ae. aegypti* populations in Franceville and, more broadly, in Gabon. These unprecedented findings reveal, for the first time, traits of resistance to pyrethroid insecticides in *Aedes* mosquito populations within the country. From a public health perspective, this study underscores the urgent need to intensify research on insecticide susceptibility in *Aedes* vectors throughout Gabon, including at a national level, to better inform control strategies. Addressing critical gaps, particularly in the characterisation of genetic mutations in the vgsc gene associated with pyrethroid resistance, as well as the metabolic mechanisms linked to resistant strains, is essential for the development of effective intervention strategies. In addition, integrated surveillance and control combining emerging genetic tools such as the sterile insect technique, biological control, environmental management, social mobilisation, and cross-sectoral collaboration [35] are promising alternatives.
